# Co-Occurrence of Microcystins and Taste-and-Odor Compounds in Drinking Water Source and Their Removal in a Full-Scale Drinking Water Treatment Plant

**DOI:** 10.3390/toxins10010026

**Published:** 2018-01-02

**Authors:** Lixia Shang, Muhua Feng, Xiangen Xu, Feifei Liu, Fan Ke, Wenchao Li

**Affiliations:** 1 State Key Laboratory of Lake Science and Environment, Nanjing Institute of Geography and Limnology, Chinese Academy of Sciences, Nanjing 210008, China; lxshangsun@163.com (L.S.); xgxuc@163.com (X.X.); feifei1845@126.com (F.L.); fke@niglas.ac.cn (F.K.); wchli@niglas.ac.cn (W.L.); 2 Key Laboratory of Marine Ecology and Environmental Sciences, Institute of Oceanology, Chinese Academy of Sciences, Qingdao 266071, China; 3 Changzhou Academy of Environmental Science, Changzhou 213022, China; 4 Nanjing Municipal Design and Research Institute CO., Ltd., Nanjing 210008, China

**Keywords:** microcystins, taste-and-odor compounds, water source, drinking water treatment plant, cyanobacterial thresholds, The co-occurrence of microcystins and taste-and-odor compounds accompanied by dominant species *Microcystis* and *Dolichospermum* were studied in a drinking water source and in the associated full-scale DWTP. Not only microcystins but also taste-and-odor compounds were taken into account to guide the management in source water and in DWTPs.

## Abstract

The co-occurrence of cyanotoxins and taste-and-odor compounds are a growing concern for drinking water treatment plants (DWTPs) suffering cyanobacteria in water resources. The dissolved and cell-bound forms of three microcystin (MC) congeners (MC-LR, MC-RR and MC-YR) and four taste-and-odor compounds (geosmin, 2-methyl isoborneol, *β*-cyclocitral and *β*-ionone) were investigated monthly from August 2011 to July 2012 in the eastern drinking water source of Lake Chaohu. The total concentrations of microcystins and taste-and-odor compounds reached 8.86 μg/L and 250.7 ng/L, respectively. The seasonal trends of microcystins were not consistent with those of the taste-and-odor compounds, which were accompanied by dominant species *Microcystis* and *Dolichospermum*. The fate of the cyanobacteria and metabolites were determined simultaneously after the processes of coagulation/flocculation, sedimentation, filtration and chlorination in the associated full-scale DWTP. The dissolved fractions with elevated concentrations were detected after some steps and the breakthrough of cyanobacteria and metabolites were even observed in finished water. Chlorophyll-*a* limits at intake were established for the drinking water source based on our investigation of multiple metabolites, seasonal variations and their elimination rates in the DWTP. Not only microcystins but also taste-and-odor compounds should be taken into account to guide the management in source water and in DWTPs.

## 1. Introduction

Cyanobacteria are well known for their ability to produce diverse secondary metabolites including microcystins (MCs) and taste-and-odor (T&O) compounds [1,2,3]. Due to the acute and potentially chronic effects of MCs, the World Health Organization [4] issued a guideline value of 1.0 μg/L for microcystin-LR (MC-LR) in drinking water. T&O compounds include geosmin (GEO) and 2-methyl isoborneol (MIB) with an earthy and musty odor, respectively, and *β*-cyclocitral (CYC) and *β*-ionone (ION) with a tobacco and violet odor, respectively [3]. These compounds are the primary barriers to drinking water safety. Due to extremely low odor threshold concentrations (OTCs) with 4, 15, 19.3 and 7 ng/L for GEO, MIB, CYC and ION, respectively [3], T&O compounds even at low levels in water are a source of complaints by consumers. Although the effects of T&O compounds on human health are still unclear, they are the primary criteria of drinking water safety considered by consumers [3], which pose challenges for drinking water treatment plant (DWTP) management.

The occurrence and seasonal dynamics of MCs or T&O compounds have been well studied in surface waters worldwide [5,6,7,8]. Recently, some researchers began to focus upon the co-occurrence of cyanotoxins and T&O compounds in lakes, reservoirs and rivers. During the 2007 summer’s odorous tap water crisis in Wuxi (China), an MC-LR of 7.59 μg/L was detected in the source water at the intake [9]. Graham et al. [10] concluded that microcystin co-occurred with geosmin in 87% of the cynobacterial blooms and MIB in 39% sampled from 23 Midwestern United States lakes. However, seasonal dynamics of MC and T&O compounds may not be so uniform due to their different producers, physicochemical properties, metabolic pathways, and environmental influencing factors [7,11]. Conflicting results in the relationship between MCs and T&O compounds with positive or no correlations were obtained in different waters [10,12], which would complicate their removal by treatment processes in DWTPs.

Scientific literature has reported that conventional water treatment techniques have been ineffective in removing MCs and T&O compounds from water [13,14]. However, most previous studies focused on the removal of algal cells and their associated metabolites only in laboratory experiments or along treatment trains, which did not always conform to reality due to a range of uncertainties in actual situations [13,15,16]. The scant literature involving cyanobacterial metabolites removal by pilot- or full-scale treatment plants present conflicting results where some plants were said to have removed MCs below the guideline of 1.0 μg/L [17], but, in another case, a MC concentration of 2.47 μg/L was detected in the drinking water [18]. Therefore, it was necessary to investigate the fate of cyanobacteria and their metabolites under typical plant operation conditions to assess the potential removal. Moreover, no studies to date have estimated the efficiency of full-scale DWTPs in removing the mixtures of MCs and T&O compounds co-occurring in the same water source, which is needed to comprehensively assess and minimize the hazards caused by cyanobacteria and their metabolites.

To estimate the safety of raw water with cyanobacteria, the Alert Levels Framework (ALF) has been established to define threshold values according to the MC-LR cell quota and the WHO guideline value of MC-LR in drinking water [2]. More than 100 MC variants have been characterized from bloom samples and isolated strains of cyanobacteria [1]; thus, the safety threshold for cyanobacteria was established based on different MC congeners at different cyanobacterial growth periods [19]. To avoid overestimating the risk of cyanobacteria in drinking source water, maximum tolerable (MT) values were calculated involving the toxin removal performance of real treatment trains by Schmidt et al. [20]. In this study, we improved these previous methods by taking account of multiple metabolites with seasonal variations and removal rates of plants and thereby pave the way for a refined management strategy designed to solve the cyanobacterial threat in DWTPs.

The objectives of the present study were to: (1) evaluate the seasonal variations of the co-occurrence of three MC congeners and four T&O compounds associated with two cyanobacterial dominant species, *Microcystis* and *Dolichospermum*, in the eastern drinking water source (EDWS) of Lake Chaohu; (2) assess the fate and elimination of cyanobacteria and the studied metabolites at each treatment step in the full-scale DWTP; and (3) establish the chlorophyll-*a* limits at intake (CLIs) for the drinking water source involving different dominant cyanobacterial species producing more than one kind of metabolites and their removal rates in the DWTP during different seasons. This research is expected to help mitigate the problems associated with cyanobacteria and their metabolites in DWTPs, which will naturally lead to safer drinking water. To the best of our knowledge, this is the first study to report the elimination of the mixtures of MCs and T&O compounds in a full-scale DWTP.

## 2. Results

### 2.1. Seasonal Variations of Cyanobacteria, MCs and T&O Compounds in the EDWS

In the EDWS of Lake Chaohu, cyanobacteria and their associated metabolites including MCs and T&O compounds were studied monthly from August 2011 to July 2012 (Table 1). The chlorophyll-*a* concentrations ranged from 5.4 to 68.0 μg/L with the lowest and highest average concentrations detected in the spring (9.0 ± 3.0 μg/L) and in the summer (38.5 ± 14.0 μg/L), respectively. The dominant phytoplankton was cyanobacteria with *Microcystis* and *Dolichospermum* making up more than 98% of the cyanobacterial abundance except in the spring (85%). Cyanobacterial density was lowest in the spring with only *Dolichospermum* detected in most samples. From summer to the middle of autumn, the *Microcystis* abundance increased considerably as the dominant species and the cyanobacteria reached a peak density of 195,000 cells/mL simultaneously. In late autumn and winter, the amount of filamentous species belonging to the genera *Dolichospermum* increased gradually and again became the dominant species with the maximum number of cyanobacteria (200,000 cells/mL) measured.

Co-occurrence of three MC congeners and four T&O compounds with different seasonal trends have been observed in this water source (Table 1). The total concentrations of the three studied MC congeners (MC-LR, MC-RR and MC-YR) ranged from 0.28 to 8.86 μg/L. In the spring, the average concentration of each MC congener was at its lowest value corresponding to the lowest cyanobacterial density of the whole year. In summer and autumn, MCs occurred at higher concentrations with *Microcystis* being dominant. The MC-LR concentration reached its maximum amount in the summer with average values of 2.80 and 3.22 μg/L for EMC-LR and IMC-LR, respectively. By comparison, the highest concentrations of MC-RR (3.73 μg/L) and MC-YR (3.58 μg/L) were both detected in autumn leading to the peak concentration of the total MCs during the study period, while the concentrations of the three other MC variants remained at high levels with a sum of 4.11 μg/L in winter. The concentrations of MCs were detected above the WHO [4] recommended guideline value in summer and autumn, and even maintained a high level of nearly 1.0 μg/L in winter.

Compared to MCs, the four studied T&O compounds showed more complex seasonal dynamics in the source water (Table 1). Furthermore, the seasonal variations of dissolved T&O compounds were inconsistent with those of particle compounds. In the spring, the maximum concentrations of *d-*GEO, *d-*CYC and *d-*ION were obtained at the lowest cyanobacterial density. In the summer, the maximum concentration of MIB was detected at the highest concentrations of both *d-*MIB (193.4 ng/L) and *p-*MIB (34.0 ng/L). Additionally, higher levels of *d-*CYC and *p-*CYC were observed at concentrations around 50 ng/L, while the peak concentration of *p-*ION (152.7 ng/L) was also recorded. In autumn, relatively low concentrations of total T&O compounds were observed but also exceeded their OTCs in some samples. In winter, the maximum concentration of CYC (125.0 ng/L) was observed with the highest *p-*CYC concentration (106.3 ng/L) detected, while the concentration of *p-*ION was up to 115.8 ng/L. The four studied T&O compounds could exceed their OTCs all the year round. During the study period, 69%, 39%, 72% and 86% of the samples for GEO, MIB, CYC and ION exceeded their corresponding OTCs, respectively.

### 2.2. Relationships among Cyanobacteria, MCs and T&O Compounds in the EDWS

**Figure 1:**
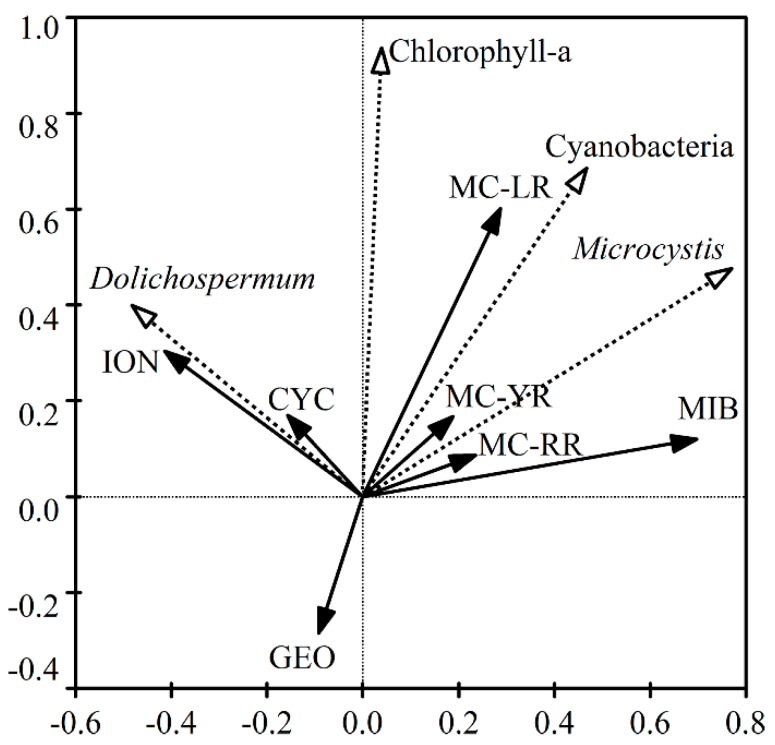
Relationships analyzed by redundancy analysis among cyanobacterial density and concentrations of microcystins and taste-and-odor compounds in the eastern drinking water source of Lake Chaohu (Intake-A, -B and -C). GEO: geosmin; MIB: 2-methyl isoborneol; CYC: *β*-cyclocitral; and ION: *β*-ionone.

Redundancy analysis was used to investigate the relationships among cyanobacteria, MCs and T&O compounds in the eastern drinking water source of Lake Chaohu (Figure 1). No relationship was found between the two predominant species *Microcystis* and *Dolichospermum*, while the density of *Microcystis* but not *Dolichospermum* was positively correlated with cyanobacterial density. Cyanobacterial density together with the *Microcystis* and *Dolichospermum* density correlated positively with the Chl-*a* concentrations. Meanwhile, the concentrations of both the MCs and T&O compounds except GEO yielded significant positive correlations with that of Chl-*a*. For these studied metabolites, strong relativities in each MC congener and MIB and positive correlations between ION and CYC concentrations were demonstrated, respectively. The concentrations of GEO had no relationship with that of ION and CYC, but correlated negatively with the rest metabolites.

### 2.3. The Removal of Chlorophyll-a, MCs and T&O Compounds via Treatment Processes in the DWTP

#### 2.3.1. Coagulation/Flocculation (C/F+)

**Figure 2:**
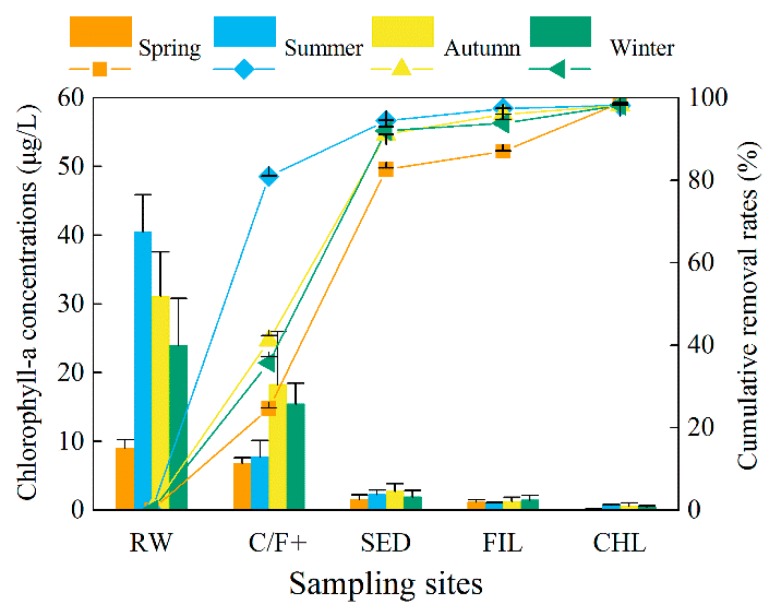
The concentrations of chlorophyll-*a* (left, column) and cumulative removal rates (right, line + scatter) in different seasons of the water treatment processes including raw water (RW), coagulation-flocculation (C/F+, with potassium permanganate and powdered activated carbon in summer and autumn), sedimentation (SED), filtration (FIL) and chlorination (CHL) in the drinking water treatment plant associated with Intake-A. Error bars indicate the standard deviation of data in three months of each season.

**Figure 3:**
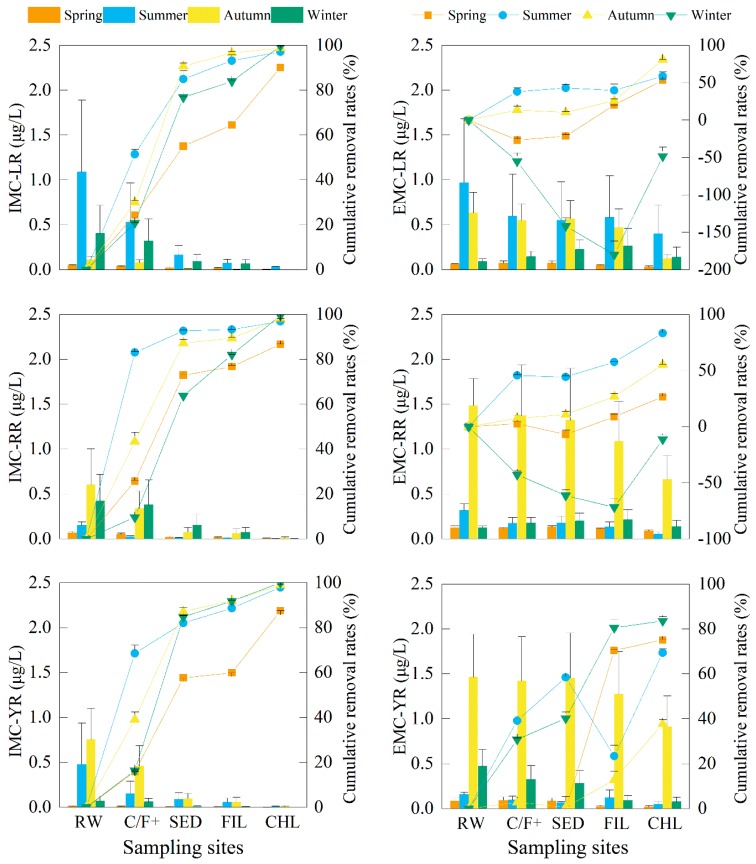
The concentrations of extra- (E) and intra-cellular (I) microcystins (MC-LR, MC-RR and MC-YR) (column) and cumulative removal rates (line + scatter) in different seasons of the water treatment processes including raw water (RW), coagulation-flocculation (C/F+, with potassium permanganate and powdered activated carbon in summer and autumn), sedimentation (SED), filtration (FIL) and chlorination (CHL) in the drinking water treatment plant associated with Intake-A. Error bars indicate the standard deviation of data in three months of each season.

**Figure 4:**
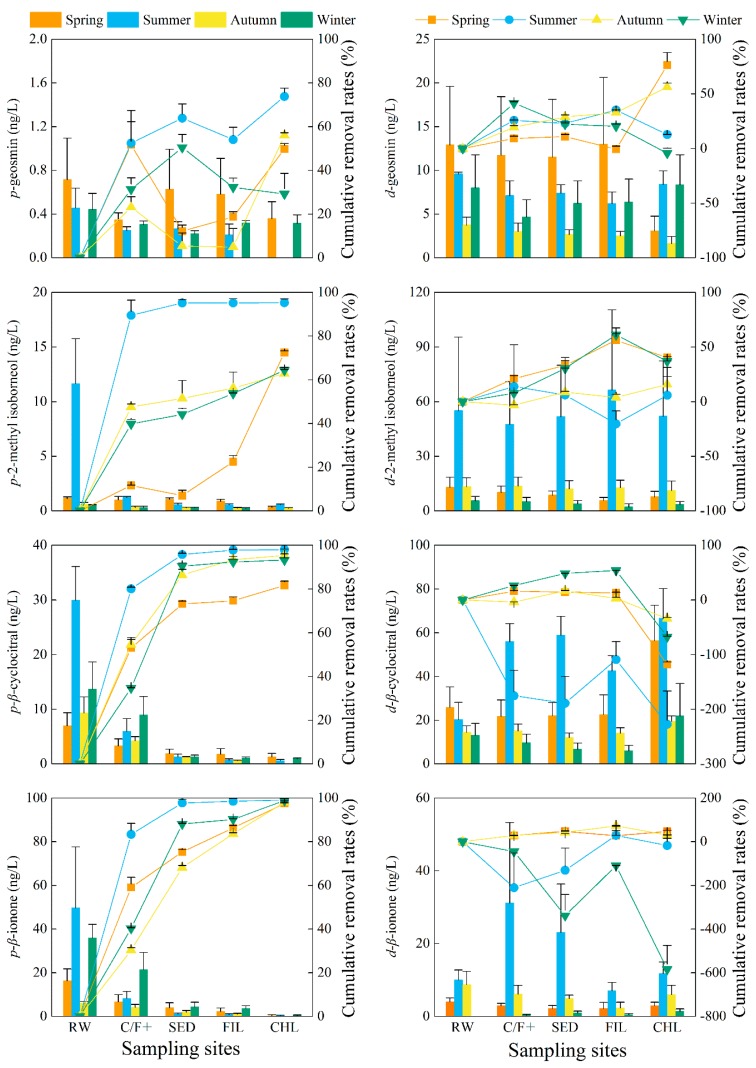
The concentrations of dissolved (*d*-) and particle-bound (*p*-) taste-and-odor compounds (column) and cumulative removal rates (line + scatter) in different seasons of the water treatment processes including raw water (RW), coagulation-flocculation (C/F+, with potassium permanganate and powdered activated carbon in summer and autumn), sedimentation (SED), filtration (FIL) and chlorination (CHL) in the drinking water treatment plant associated with Intake-A. Error bars indicate the standard deviation of data in three months of each season.

The concentrations of cyanobacteria and the associated metabolites in raw water sampled from the DWTP showed no significant differences from that sampled at Intake-A in the studied EDWS (*p* > 0.05). As the first step to remove algae and metabolites, the Coagulation/Flocculation effects must be assessed to ensure minimal impacts on the subsequent processes and improve total removal efficiencies of DWTP. In this study, the elimination efficiency of chlorophyll-*a* from raw water to the C/F+ (+, with 9 mg/L potassium permanganate (KMnO_4_) and 10 mg/L powdered activated carbon (PAC) in summer and autumn with contact time of 30 min) process ranged from 24.6 to 80.9% (45.6% average) (Figure 2). The removal rates of intracellular MCs and T&O compounds ranged from 9.7 to 89.5% (43.8% average) (Figure 3 and Figure 4). The maximum removal rates of chlorophyll-*a* and the cell-bound metabolites by the C/F+ process were both observed in summer. The elimination rates of the dissolved MCs and T&O compounds were lower than 45.7%, and the concentrations of some dissolved metabolites even increased after the C/F+ step. The concentrations of EMC-LR, EMC-RR and *d-*ION increased by 55.2%, 42.8% and 45.3% (on average) in winter, respectively, while those of *d-*CYC and *d-*ION increased by 175.1% and 210.7% in summer, respectively.

#### 2.3.2. Sedimentation (SED)

The sedimentation step in DWTP is used to settle the particles including cyanobacterial flocs in suspension into the sediment and out of the fluid. More than 90% of the chlorophyll-*a* were further removed by sedimentation except in the spring (82.7%), which made up 13.8–58.8% of total removal efficiency in the studied DWTP. Meanwhile, 50.4–97.8% of the three IMC congeners, *p*-CYC and *p*-ION were further removed by the sedimentation step. However, the concentrations increased after sedimentation for *p-*GEO in the spring and autumn and *p-*MIB in the spring. For dissolved metabolites, the sedimentation efficiencies were less than 30.2% compared to the concentrations in the C/F+ effluent. Furthermore, the concentrations of EMC-LR, EMC-RR, *d*-GEO and *d*-CYC increased by 55.7%, 13.0%, 33.1% and 201.7% compared to those in the C/F+ effluent in winter, respectively. In the spring, summer and autumn, the dissolved metabolites in 38.1% of the studied samples increased in a range of 1.6–9.9% compared to those metabolites in the effluent of C/F+ step.

#### 2.3.3. Sand Filtration (FIL)

Sand filtration is primarily used to remove the excess flocs after sedimentation. In this step, the elimination of chlorophyll-*a* represented less than 5% of the total removal rate of the whole processes in the DWTP. In filtered water, the average concentration of chlorophyll-*a* was 1.2 ± 0.2 μg/L. Compared to the concentrations in the sedimentation effluent, 5.7–63.6% (32.9% on average) of the cell-bound metabolites were reduced further by filtration. With an average removal rate of 14.0%, the concentrations of extracellular metabolites increased in 39.2% of the samples after filtration compared to that of in the sedimentation effluent, where summertime rates for EMC-YR and *d*-MIB increased by 84.5% and 28.2%, respectively.

#### 2.3.4. Chlorination (CHL)

Post-treatment chlorination provides the last defense against cyanobacterial cells, MCs, and T&O compounds reaching consumers in water treated by the DWTP. In this study, the chlorophyll-*a* removal rates by chlorination were between 30.1% and 90.2% compared to the concentrations in the filtration effluent. After chlorination, the maximum concentration of each intracellular MC and T&O compound was 0.03 μg/L (IMC-LR in the summer) and 1.9 ng/L (*p*-CYC in the spring), respectively. For extracellular MCs, the chlorination process further reduced the concentrations from 15.7 to 74.2% compared to those in the filtration effluent. However, the concentration of each T&O compound increased from 31.0 to 266.7% after chlorination except for *d*-GEO in the spring and autumn and *d*-MIB in the summer and autumn, respectively.

In the finished water, the average concentrations of chlorophyll-*a* for each season were between 0.1 ± 0.0 μg/L and 0.7 ± 0.1 μg/L. The maximum concentrations of MC-LR, MC-RR and MC-YR in the finished water were 0.4 ± 0.3 μg/L (in the summer), 0.7 ± 0.3 μg/L (in the autumn) and 0.9 ± 0.3 μg/L (in the autumn), respectively. The concentrations of T&O compounds varied within a wider range compared to those of MCs in the drinking water. The concentrations of GEO above its OTC of 4 ng/L were detected in the summer (8.4 ± 1.6 ng/L) and winter (8.7 ± 3.5 ng/L). The CYC exceeded its OTC throughout the whole year with concentrations ranging from 19.5 ± 2.4 ng/L to 67.2 ± 13.8 ng/L. In the summer, the concentrations of MIB and ION were 52.5 ± 30.5 ng/L and 12.2 ± 3.3 ng/L, respectively. Both readings exceeded their corresponding OTC standard.

#### 2.3.5. Total Removal Rates of Chlorophyll-*a* and the Metabolites

**Figure 5:**
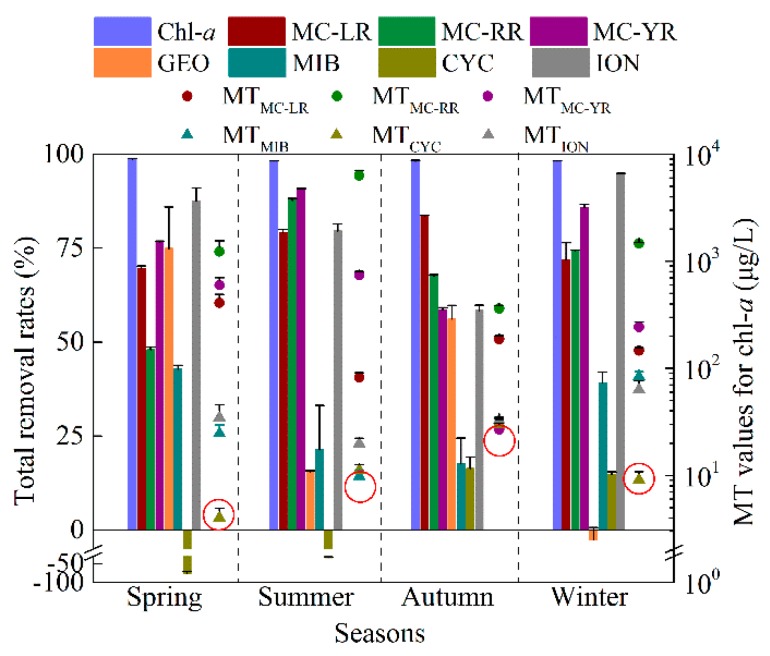
The removal rates of chlorophyll-*a* and the microcystins and taste-and-odor compounds in the drinking water treatment plant associated with Intake-A (left) and the maximum tolerable (MT) values (right) based on MC-LR, MC-RR, MC-YR, 2-methyl isoborneol (MIB), *β*-cyclocitral (CYC) and *β*-ionone (ION); the establishment of chlorophyll-*a* (Chl-*a*) limits at intake (CLIs) in the four seasons in the eastern drinking water source of Lake Chaohu (red circle). Error bars indicate the standard deviation of total removal rates in different months of each season and the MT values for Chl-*a* of three sampling sites.

The seasonal average of removal efficiencies of chlorophyll-*a* and the studied metabolites are shown in Figure 5. On average, more than 98% of chlorophyll-*a* was removed from water. The total removal rates of the MC-LR, MC-RR and MC-YR varied from 47.9 to 90.9% with an average of 74.5%. For T&O compounds, the average removal rates during the whole year for GEO and MIB were 36.0% and 30.2%, respectively. For CYC, the removal efficiencies of 16.3% and 14.8% were detected in the autumn and winter, respectively. However, the concentrations of CYC were increased by 75.7% and 33.7% in the finished water compared to the concentrations in the raw water in the spring and summer, respectively. The ION removal rates were between 58.4% and 94.9% with an average of 80.1% during the whole year.

## 3. Discussion

### 3.1. The Co-Occurrence of MCs and T&O Compounds Relating to Different Dominant Cyanobacterial Species in the EDWS

In the EDWS of Lake Chaohu, the co-occurrence of MCs and T&O compounds caused adverse effects on the safety of water source. Previously, different conclusions were drawn involving the relationships between MCs and T&O compounds. For particulate T&O compounds, significant correlations between *p-*CYC and *p-*ION with MCs were reported in Lake Taihu [12]. However, no correlation between toxins and T&O compounds concentrations was found in the survey involving 23 Midwestern United States lakes [10]. When two cyanobacterial species, *Microcystis* and *Dolichospermum*, dominated in different seasons, dynamic changes relating to these metabolites became vastly more complicated compared to the water source with the sole dominant species. In the present study, strong relativities in each MC congener and MIB were found and positive correlations between ION and CYC were demonstrated (Figure 1). The GEO had no relationship with ION and CYC, but correlated negatively with the remaining metabolites. The mutable relativities among the three MCs congeners and four T&O compounds could be explained by the community dynamics of synthesizing cyanobacterial strains, different metabolic pathways, and the different physicochemical properties such as volatility and degradability.

The DWTPs usually reinforce treatment technology operation during cyanobacterial blooming at warmer temperatures instead of operating within a dedicated system year round from the perspective of cost savings [16]. However, in the drinking water sources associated with more than one dominant cyanobacterial species such as those found in this study, the concentrations of MCs and T&O compounds still maintained at high levels in spring and winter and even exceeded the guidelines during the whole year. The differences in the characteristics and seasonal variations of the two classified compounds (MCs and T&O) indicated the high risk involved in producing safe drinking water, which has led to difficult and complex management for the drinking water source and the DWTP.

### 3.2. The Fate of Chlorophyll-a, MCs and T&O Compounds in the Associated DWTP

In the studied DWTP, the removal efficiencies of the cyanobacteria and associated metabolites varied with seasons and depended on several factors including physicochemical characteristics of metabolites, community dynamics of dominating cyanobacterial species and the mode of operation in the DWTP.

As the primary step to remove pollutants, the C/F+ process gained more effective removal in summer and autumn for chlorophyll-*a* and the cell-bound metabolites compared to that in spring and winter. According to the coagulation mechanism of charge neutralization [13], spherical aggregates of *Microcystis*, which are dominate in summer and autumn, are easier to remove compared to the filamentous *Dolichospermum*, which is dominate in spring and winter. Fan et al. [15] found that the use of KMnO_4_ in summer and autumn could improve algae coagulation and the removal of cell-bound metabolites by reducing cell stability and inducing cell decay. Ghernaout et al. [13] found the C/F+ step to be inefficient for removal of soluble organics in water, which was further demonstrated by the low removal rates of the dissolved cyanobacterial metabolites in the present study. Even more, increased concentrations of dissolved metabolites after the C/F+ process were measured in half of the samples compared to their concentrations in the raw water. In the winter, increasing concentrations of EMC-LR, EMC-RR and *d*-ION were observed with their cell-bound forms dominating the total concentrations. Therefore, cells lysis and metabolite release occurred due to shear stress in the C/F step aiming to quickly and uniformly disperse coagulants and form flocs [13,21]. However, only the concentrations of *d-*CYC and *d-*ION increased dramatically during the summer, although the intracellular concentrations of MCs and T&O compounds both had high concentrations. This indicated that the release rates of intracellular CYC and ION from damaged cells were faster than that of degradation in the C/F process or by chemicals such as KMnO_4_ dosed in the reaction tank. The inconsistent performance of MCs with T&O might be because of the differences in the two classes of metabolites in molecular structure, physicochemical characteristics, and release and degradation rates such as the more volatile components of the odorous compounds.

Most suspended particles and flocs with algal cells formed in coagulation can be removed by sedimentation through gravity precipitation. The elimination efficiencies of the chlorophyll-*a* and the cell-bound metabolites except for *p-*GEO were more than 81% during the treatment train and most of them were removed by the C/F+ and sedimentation steps, which were in accordance with the data (62.0–98.9%) summarized by Zamyadi et al. [18]. However, a rising level in dissolved fraction after sedimentation was observed for both MCs and T&O compounds during the whole year especially in winter with no further treatment used. The sludge in the sedimentation tank became a potential source of organic matter as a storage deposit of flocs with treated cyanobacterial cells after the C/F process [22]. Moreover, GEO and MIB could also be produced by benthic microorganisms growing in the sludge bed other than phytoplankton [23]. Therefore, the sludge with cyanobacteria cells from the sedimentation process should be removed rapidly and safely disposed of to prevent additional hazards caused by cyanobacterial cell lysis.

Cell breakthroughs were observed after filtration in the studied DWTP, with an average chlorophyll-*a* concentration of around 1.2 μg/L in the filtered water. In addition, an increase of extracellular metabolites was observed, such as EMC-YR and *d*-MIB in summer. The cyanobacteria accumulated on the filters and biofilm fragments or other impurities attached to filters might cause subsequent organics to be released into the treatment plant water [21]. Hence, it is necessary to increase backwash time and frequency to improve the filtration efficiency in the removal of cyanobacteria and the associated metabolites.

The chlorination process is generally regarded as a step to reduce organic matter, which can breed harmful bacteria once released into the water distribution network. Incomplete removal of cyanobacterial metabolites by the preceding treatment processes requires more chlorine to guarantee enough residual chlorine in the water distribution network. Serious safety problems could occur if disinfection byproducts (DBPs) are formed in drinking water. Although the chlorination step was effective to remove three MCs congeners, the concentrations of T&O compounds increased after chlorination, especially for *d-*CYC and *d-*ION. This phenomenon contradicts the results found by Zhang et al. [24] that CYC and ION could be removed by chlorination if the process followed a pseudo first-order and a pseudo second-order kinetics mechanism, respectively. However, their previous kinetics experiments using pure odorant chemicals diluted in ultrapure water were not disrupted by other competitive organic compounds such as that found in natural (raw) water. Höckelmann and Jüttner [25] found that CYC forms by an oxidative cleavage reaction of *β*-carotene and is catalyzed by *β*-carotene oxygenase bound on *cyanobacterial* cells under aerobic conditions; thus, the results in this study might be because chlorination induced the production of CYC and ION via conversion reactions of carotenoids, which coincided with the increased CYC observed in the oxidation of *β*-carotene with permanganate oxidation [26]. Further investigation is needed to demonstrate the reaction mechanism to reveal the difference between the laboratory scale chlorination experiments and the operational conditions of raw water suffering from off-flavor problem.

### 3.3. The Establishment of Chlorophyll-a (Chl-a) Limits at Intake (CLIs) for DWTP and Reservoir Management

The conventional DWTP was effective for removing cyanobacterial cells but not for the dissolved cyanobacterial metabolites. Together with the release of metabolites by cyanobacterial cells in the C/F process and accumulated in sludge and filtering material, the dissolved organic matter could not be removed effectively before chlorine dosing disinfection generated DBPs [27]. Therefore, controlling cyanobacteria in a water source or before entering the DWTP is important to guarantee the safety of drinking water.

In this study, the concentrations of metabolites including three MC categories (MC-LR, MC-RR and MC-YR) and three T&O compounds (MIB, ION and CYC) significantly correlated with chlorophyll-*a* (Figure 1) and were used to calculate the CLIs for the DWTP associated with the EDWS. Correspondingly, the GEO was not chosen in the CLI system since no positive correlation was detected between GEO and algae. According to our previous paper, the guideline ($G_{met}$) of the MC-LR, MC-RR and MC-YR was 1.0 μg/L, 1.0 μg/L and 10.0 μg/L, respectively [19]. The OTCs of MIB, ION and CYC were used as their guideline ($G_{met}$), respectively. The MT values for the six chosen metabolites in each season were calculated according to their removal rates in the studies as noted by Equations (2)–(5). The CLI for each season was the minimum value of calculated MTs. Finally, the CLIs were 4.0 ± 0.9, 9.8 ± 1.4, 26.9 ± 3.9 and 9.1 ± 1.7 μg/L according to the MT value of CYC, MIB, MC-YR and CYC in the spring, summer, autumn and winter, respectively (Figure 5). These values were between the threshold of 1 and 50 μg/L chlorophyll-*a* for the Alert Level 1 and 2 [2]. The relatively low CLIs obtained in the present study were not only determined according to MC-YR in autumn but also according to T&O compounds in other seasons. It is worth noting that the CLIs might be unusually low for the DWTP which has been in operation for more than 30 years. The sludge and filters in the sedimentation and filtration step have accumulated a large amount of cyanobacteria, microorganisms and other organic contaminants which resulted in increasing concentrations of dissolved microcystins and T&O in the effluent. Consequently, low removal rates are obtained and then low CLIs are required based on the condition of the DWTP.

Compared to the cyanobacterial density or biovolume, chlorophyll-*a* concentrations were easier to detect and more suitable to be used as the indicated index especially for the water sources suffering with different algal species and many variants of cyanotoxins [1,28]. This method to establish chlorophyll-*a* thresholds in different seasons is not only based on MCs but also on T&O compounds to guide the management of the water source and the DWTP and to guarantee the safety of drinking water involved in multiple contaminants. Because the water sources are specific with different water quality standards and cyanobacterial species and because DWTPs each have unique treatment technologies, we recommend that water companies establish their own standards through routine monitoring of water source quality using the proposed evaluation system. Considering the possibilities of forming DBPs after chlorination with the remnant organic matter in the preceding processes, the DBPs and their precursors should also be taken into account for the reservoir and DWTP management.

## 4. Conclusions

In the EDWS of Lake Chaohu, three MC congeners and four T&O compounds co-occurred in different seasonal dynamics with two cyanobacterial dominant species, *Microcystis* and *Dolichospermum*. The MCs exceeded the WHO [4] recommended guideline value in summer and autumn and the T&O compounds were above their OTCs throughout the whole year except for MIB in winter, which complicated the management of DWTP and posed great risk on drinking water production. The cell-bound metabolites were removed mainly by the C/F+ step with the elevated elimination observed in summer and autumn in the associated full-scale DWTP with water treatment techniques including C/F (with potassium permanganate and powdered activated carbon in summer and autumn), sedimentation, filtration and final chlorination. However, an increasing concentration of dissolved metabolites were observed along with the treatment techniques, especially after C/F+ and chlorination, resulting in some T&O compounds exceeding their corresponding OTCs in the finished water. Due to the co-occurrence of the two classes of cyanobacterial metabolites with different characteristics, CLIs synthesis during their seasonal variations and their elimination rates were proposed to help managers make proper strategies in source water and DWTPs to guarantee the safety of drinking water.

## 5. Materials and Method

### 5.1. Chemicals, Standards and Materials

Microcystin standards (MC-LR, MC-RR and MC-YR) with purities ≥95% were purchased from Alexis Biochemicals (Lausen, Switzerland). The stock solution of three MC congeners was prepared in 1 mL of methanol solution at a concentration of 100 mg/L for each variant, which was kept at −20 °C for later use. The C18 solid phase extraction (SPE) cartridges (500 mg, 6 mL) used in MCs analysis were obtained from Anpel Company (Shanghai, China). The standard compounds GEO, MIB and 2-isobutyl-3-methoxypyrazine (IBMP, as the internal standard) were purchased from Sigma-Aldrich Chemical Co. (St. Louis, MO, USA) with the concentration of 100 mg/L in methanol, while CYC and ION with purities ≥97% were purchased from Adamas Reagent Co., Ltd. (Basel, Switzerland). The stock solution of 1 mg/L with four target T&O compounds prepared in methanol was stored in the dark at 4 °C. Sodium chloride (NaCl, Sigma, St. Louis, MO, USA) was added to the samples to enhance the T&O compounds extraction from water.

Methanol, trifluoracetic acid, acetic acid, acetonitrile and acetone were of HPLC grade from Tedia Company (Fairfield, OH, USA). Water used throughout the work was from a Milli-Q water purification system (Millipore, Billerica, MA, USA). All other reagents used were of analytical grade or better.

### 5.2. Study Site

**Figure 6:**
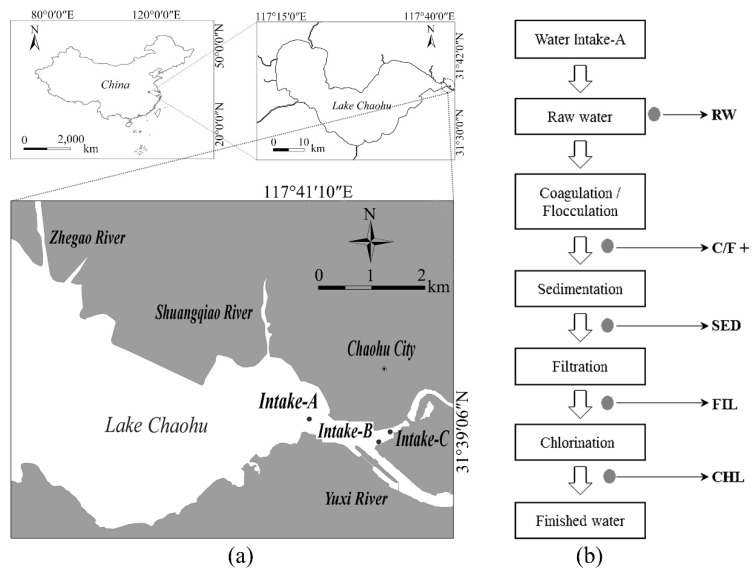
Sampling sites: (**a**) in the eastern drinking water source of Lake Chaohu; and (**b**) in the drinking water treatment plant associated with Intake-A.

Lake Chaohu, the fifth largest shallow lake in China, has four distinct seasons influenced by East Asia monsoons with multiple uses such as water supply, commercial fishery and sightseeing. Eastern Lake Chaohu is the sole drinking water source for around 4.23 million people in the city of Chaohu. To evaluate the impact of cyanobacteria and their metabolites on the safety of EDWS, we took three main drinking water intakes at EDWS as sampling sites: Intake-A (117°50′15.13″ E, 31°35′36.34″ N), Intake-B (117°50′54.32″ E, 31°35′24.82″ N) and Intake-C (117°50′58.40″ E, 31°35′32.30″ N) (Figure 6a). The seasonal variations of physicochemical parameters in this drinking water source during the sampling period are summarized in Table 1.

The full-scale DWTP associated with Intake-A was chosen to evaluate the elimination of cyanobacteria and their metabolites in treatment processes with the routine sequences of coagulation-flocculation (C/F), sedimentation, filtration and chlorination. The production rate of the DWTP was 40,000 m^3^/d. Water from Lake Chaohu was transported through a water pipe at a distance of 2 km. In general, the dosing of coagulant polyaluminum ferric chloride and flocculation aid dimethyl diallyl ammonium chloride were 12 mg/L and 2 mg/L, respectively. In summer and autumn, 9 mg/L of KMnO_4_ and 10 mg/L of PAC were dosed into a reaction tank with coagulants and flocculants.

### 5.3. Sampling, Sample Preparation and Phytoplankton Analysis

Water samples were collected monthly from August 2011 to July 2012 at the sampling stations. Integrated water samples were taken from the EDWS of Lake Chaohu (Intake-A, -B and -C), using a 2.5 L sampler. These samples were taken from the entire water column at 0.5 m intervals. Simultaneously, sampling in the DWTP was done throughout the treatment processes and included raw water (site RW), the outflows of the process of C/F (site C/F), sedimentation (site SED), filtration (site FIL) and chlorination (site CHL) (Figure 6b).

The samples for phytoplankton analysis (1 L) were fixed in situ with 1 to 1.5% Lugol’s iodine solution. The samples for the chlorophyll-*a* analysis, MC analyses and T&O compounds (without headspace) analysis were stored in 1 L high density polyethylene bottles and sealed with screw caps, respectively. These samples were placed in the dark on ice and transported back to the lab for analysis. Sodium thiosulfate was added into the MC and T&O compounds analysis samples of finished water in the field to prevent further oxidation with free chlorine [29].

Phytoplankton samples were analyzed according to taxonomic keys based on cell structure and dimension [30] using a phase contrast microscope (Nikon, TS100F, Tokyo, Japan). Chlorophyll-*a* concentrations were analyzed by a spectrophotometric method according to Jeffrey and Humphrey [31].

### 5.4. MCs Extraction and Analysis

Three MCs variants including MC-LR, -RR and -YR with extracellular forms (EMC-LR, EMC-RR and EMC-YR) and intracellular forms (IMC-LR, IMC-RR and IMC-YR) were extracted and condensed from water samples using the method modified according to Barco et al. [32]. Simply, the glass microfiber filter (GF/C, Whatman, Whatman Inc., Clifton, NJ, USA) was used to separate extra- and intra-cellular MCs. The samples of filtered water were concentrated on the SPE cartridges. After freeze-thawed three times, the filters with intra-cellular MCs were extracted using 5% acetic acid once, followed by 75% methanol twice with ultrasonic treatment prior to pre-concentration over the SPE cartridges. Before high performance liquid chromatography (HPLC) analysis, the extracts were stored at −20 °C. The identify and quantity of three MC congeners were determined by HPLC (Agilent 1200, Agilent Technologies, Santa Clara, CA, USA) with quaternary pump (G1311A), autosampler (G1329A), thermostated column compartment (G1316A), diode-array detector (G1315D), and an Agilent Eclipse XDB-C 18 column (4.6 × 150 mm i.d.; 5 µm particle size). Gradient elution of water/0.05% trifluoracetic acid (A) and acetonitrile (B) was used by varying the volume percentage of B from 30 to 40% over 15 min, and held constant for an additional 5 min. Injection volume was 20 μL, and chromatograms were analyzed and integrated at 238 nm. Microcystins in the samples were compared with MC-LR, MC-RR and MC-YR standards based on peak areas and retention times. After concentrating the samples, the detection limits for the three MC congeners in intra- and extra-cellular forms were at the 0.01 μg/L level.

### 5.5. T&O Compounds Analysis

Four T&O compounds including dissolved (*d-*GEO, *d-*MIB, *d-*CYC and *d-*ION) and particulate T&O compounds (*p-*GEO, *p-*MIB, *p-*CYC and *p-*ION) were analyzed using solid-phase microextraction followed by GC-MS which was modified according to the method of Watson et al. [33]. One liter or more water samples were filtered immediately through a Whatman GF/C glass fiber filter and divided into a dissolved and a particle-bound fraction. The filtrate (80 mL) with the dissolved fraction was transferred into 125 mL vials immediately. The filter residue with the particulate fraction was rinsed into a 125 mL vial with 80 mL of Milli-Q water, and then the cells were disrupted using ultrasound in ice bath. The sample with particle-bound T&O compounds were salted out with 25 g NaCl to improve recovery, stirred vigorously, and extracted at 65 °C for 30 min using a 2 cm long CAR/DVB/PDMS fiber (Supelco). GC-separation was done using HP 6890/5973 GC-MS equipped with a DB-5MS column. The GC was programmed from 60 °C (constant temperature for 2 min) to 200 °C (8 °C/min) with a 2 min hold, and finally to 260 °C (15 °C/min). For the selected ion monitoring mode, *m*/*z* 107 and 95 for MIB, *m*/*z* 112 and 126 for GEO, *m*/*z* 137 and 152 for CYC and *m*/*z* 177 and 91 for ION were used. Quantities of each analyte were compared to the corresponding standard curve. The detection limits were 0.2, 0.2, 0.5 and 0.4 ng/L for GEO, MIB, CYC and ION, respectively.

### 5.6. Statistical Analysis

According to the climate feature of Lake Chaohu, the sampling time was divided into four seasons: spring (March–May), summer (June–August), autumn (September–November) and winter (December–February). In the EDWS of Lake Chaohu, the data listed in Table 1 were expressed with the average value and the range of three sampling sites (Intake-A, -B and -C) for each parameter in the corresponding season. Using the multivariate data analysis software CANOCO 4.5 (Microcomputer Power, Ithaca, NY, USA) [34], redundancy analysis (RDA) was performed to determine the relationship among cyanobacteria, three MC variants and four T&O compounds. The two-tailed student’s *t* test was performed to compare the differences between data from Intake-A in the drinking water source and data from site RW in the DWTP, wherein no differences were accepted as significant at *p* > 0.05. During the removal processes, the values of the parameters in each season were presented using average data and standard deviation.

### 5.7. Calculation of Removal Rates and CLIs

The removal rates of cyanobacteria and the associated metabolites in the DWTP were calculated using average concentration of targeted pollutants in each season as:

(1) $$\eta_{i}=\left(c_{i0}-c_{i}\right)/c_{i0}\times 100\%$$

where η is the removal rate of all corresponding compounds designated by its subscript i, which represents chlorophyll-*a*, cyanobacteria and each metabolite. $c_{i0}$ and $c_{i}$ are the average concentration of chlorophyll-*a*, cyanobacteria and each metabolite measured in each season in the effluent from the preceding step $c_{i0}$ and from this treatment step $c_{i}$. Total removal rates were obtained when $c_{0}$ and c are the concentrations in the raw water and the finished water, respectively. The values with no detections or below limit of quantitation were set equal to zero for the calculations.

To ensure the safety of drinking water, the CLIs were calculated using Equations (2)–(5):

(2) $$CLI=Min\;(MT_{1},\;MT_{2},\;MT_{3},\;\ldots \ldots )$$

(3) $$MT=G_{Chl-a}/\left(1-\eta_{met}\right)$$

(4) $$G_{Chl-a}=G_{met}/c^{\prime }{}_{met}$$

(5) $$c^{\prime }{}_{met}=c_{met}/c_{Chl-a}$$

where CLI represents the chlorophyll-*a* limits at intake; MT is the maximum tolerable value for chlorophyll-*a* in the drinking water source, which was modified according to Schmidt, Bornmann, Imhof, Mankiewicz and Izydorczyk [20]; and $\eta_{met}$ is the total removal rates of the targeted metabolites in the DWTP. $G_{Chl-a}$ is the chlorophyll-*a* concentration equivalent to the guideline standard of the studied metabolites [19]. $G_{met}$ is the guideline standards or OTCs of the studied metabolites’ concentration in the drinking water. $c^{\prime }{}_{met}$ is the cell quota of metabolites, e.g., in pg per cell; $c_{met}$ is the metabolite concentration in water; and $c_{chl-a}$ is the chlorophyll-*a* concentration in water.

## Figures and Tables

**Table 1:** Mean and ranges of chlorophyll-*a*, phytoplankton density, extra- (E) and intra-cellular (I) microcystins and dissolved (*d*-) and particle-bound (*p*-) taste-and-odor compounds in the eastern drinking water source of Lake Chaohu (including Intake-A, B and C) during four seasons.

	Spring	Summer	Autumn	Winter
Chlorophyll-*a* (μg/L)	9.0 (5.4–13.7)	38.5 (22.2–68.0)	22.2 (8.0–55.7)	24.6 (5.7–47.9)
Phytoplankton density (×10^4^ cells/mL)	1.7 (0.5–4.0)	19.1 (18.0–20.2)	12.7 (5.7–17.2)	12.2 (7.0–22.1)
Cyanobacterial density (×10^4^ cells/mL)	1.5 (0.3–3.9)	18.5 (18.6–19.5)	12.5 (5.4–17.0)	12.0 (6.7–21.9)
*Microcystis* density (×10^4^ cells/mL)	0.1 (*n.d.*–0.3)	11.6 (2.8–16.5)	8.8 (3.0–13.1)	2.3 (0.9–4.8)
*Dolichospermum* density (×10^4^ cells/mL)	1.4 (0.2–3.8)	2.8 (0.6–5.2)	1.6 (1.1–2.2)	9.4 (5.7–15.5)
EMC-LR (μg/L)	0.05 (0.03–0.06)	0.98 (0.07–2.80)	0.65 (0.07–1.04)	0.14 (0.02–0.55)
IMC-LR (μg/L)	0.04 (0.02–0.08)	1.15 (0.12–3.22)	0.11 (0.03–0.18)	0.36 (0.04–1.31)
EMC-RR (μg/L)	0.12 (0.06–0.17)	0.31 (0.21–0.59)	1.42 (0.08–2.45)	0.12 (0.06–0.22)
IMC-RR (μg/L)	0.07 (0.05–0.11)	0.16 (0.11–0.25)	0.56 (0.03–1.47)	0.41 (0.10–1.20)
EMC-YR (μg/L)	0.07 (*n.d.*–0.12)	0.18 (0.11–0.27)	1.34 (0.21–2.83)	0.46 (*n.d.*–0.74)
IMC-YR (μg/L)	0.01 (*n.d.*–0.02)	0.37 (0.02–1.07)	0.82 (0.05–1.35)	0.11 (0.01–0.31)
*d*-geosmin (ng/L)	16.8 (1.6–47.8)	8.1 (4.8–12.1)	3.7 (1.3–7.2)	7.7 (*n.d.*–17.2)
*p*-geosmin (ng/L)	0.7 (0.2–1.7)	0.6 (0.2–1.1)	*n.d.*	0.4 (*n.d.*–0.9)
*d*-2-methyl isoborneol (ng/L)	11.1 (4.5–30.7)	60.2 (*n.d.*–193.4)	14.1 (0.7–27.5)	5.4 (*n.d.*–11.8)
*p*-2-methyl isoborneol (ng/L)	1.3 (0.4–2.0)	12.6 (0.6–4.0)	0.5 (*n.d.*–1.3)	0.5 (*n.d.*–1.7)
*d*-*β*-cyclocitral (ng/L)	26.5 (8.0–50.1)	21.9 (8.4–49.2)	14.9 (8.2–33.8)	10.6 (*n.d.*–20.0)
*p*-*β*-cyclocitral (ng/L)	4.7 (1.0–10.9)	26.3 (5.4–53.4)	4.0 (0.9–8.7)	42.5 (5.4–106.3)
*d*-*β*-ionone (ng/L)	5.8 (0.4–28.4)	10.8 (3.3–23.2)	7.3 (*n.d.*–21.8)	0.4 (*n.d.*–1.1)
*p*-*β*-ionone (ng/L)	14.5 (1.8–30.1)	53.4 (2.6–152.7)	4.8 (0.7–11.6)	47.1 (3.5–115.8)

*n.d.*: below the lower-limit of the calibration range.
